# The Role of Mitochondrial Dysfunction and ER Stress in TDP-43 and C9ORF72 ALS

**DOI:** 10.3389/fncel.2021.653688

**Published:** 2021-04-01

**Authors:** Ruxandra Dafinca, Paola Barbagallo, Kevin Talbot

**Affiliations:** Nuffield Department of Clinical Neurosciences, University of Oxford, Oxford, United Kingdom

**Keywords:** C9orf72, TDP-43, calcium homeostasis, ALS, mitochondria, endoplasmic reticulum, UPR

## Abstract

Amyotrophic lateral sclerosis (ALS) is a neurodegenerative disease of the motor system with complex determinants, including genetic and non-genetic factors. Despite this heterogeneity, a key pathological signature is the mislocalization and aggregation of specific proteins in the cytoplasm, suggesting that convergent pathogenic mechanisms focusing on disturbances in proteostasis are important in ALS. In addition, many cellular processes have been identified as potentially contributing to disease initiation and progression, such as defects in axonal transport, autophagy, nucleocytoplasmic transport, ER stress, calcium metabolism, the unfolded protein response and mitochondrial function. Here we review the evidence from *in vitro* and *in vivo* models of C9ORF72 and TDP-43-related ALS supporting a central role in pathogenesis for endoplasmic reticulum stress, which activates an unfolded protein response (UPR), and mitochondrial dysfunction. Disruption in the finely tuned signaling between the ER and mitochondria through calcium ions may be a crucial trigger of mitochondrial deficits and initiate an apoptotic signaling cascade, thus acting as a point of convergence for multiple upstream disturbances of cellular homeostasis and constituting a potentially important therapeutic target.

## Introduction

Amyotrophic lateral sclerosis (ALS) is the most common type of motor neuron diseases and is characterized by progressive degeneration of upper and lower motor neurons, resulting in loss of voluntary muscle action and eventually death through respiratory failure. While the majority of cases are sporadic, ~10% of ALS is due to inheritance of a genetic mutation in an autosomal dominant pattern. To date, mutations in up to 20 genes have been associated with ALS, including superoxide-dismutase 1 (SOD1), TAR-DNA binding protein (TARDBP) and FUS (Mathis et al., 2019; McCann et al., 2020). However, the most common single mutation is a GGGGCC hexanucleotide repeat expansion in the C9ORF72 gene, which accounts for ~40% of all familial ALS and significant numbers of apparently sporadic cases, and provides a clear genetic link to frontotemporal dementia (FTD) (DeJesus-Hernandez et al., 2011; Renton et al., 2011).

There are multiple potential mechanisms through which the hexanucleotide might drive pathogenesis in C9ORF72-related disease, leading to either toxic gain of function or loss of normal function: formation of nuclear RNA foci that sequester a range of hnRNPs; production of poly-dipeptides (GA, GP, GR, PA, PR) through repeat non-ATG (RAN) translation leading to the accumulation of toxic aggregates; or haploinsufficiency due to transcriptional silencing. While these mechanisms are not mutually exclusive, and each may operate in different phases of disease, the relative contribution of acquired toxicity and loss of normal function as initiating factors in C9ORF72-related neurodegeneration is still unclear.

Despite the genetic heterogeneity of familial ALS, abnormal accumulation of misfolded or aggregated proteins is a unifying pathological feature. The organelle mainly responsible for native folding, post-translational modifications and trafficking of many proteins is the endoplasmic reticulum (ER) (Schroder, 2008; Bernard-Marissal et al., 2015b). Quality control performed by ER-resident chaperones ensures precise folding of newly synthesized proteins and identifies unfolded or misfolded proteins, which are then targeted to specific degradation pathways (Kaushik and Cuervo, 2015). When proteostasis is disturbed through accumulation of misfolded or unfolded proteins, a stress response is triggered which is mitigated by an adaptive signaling mechanism called the “unfolded protein response” (UPR). The UPR reduces general translation and enhances the expression of specific UPR target genes, such as ER chaperones to restore protein folding and promote quality control mechanisms or degradation of irreversible misfolded proteins. Dysfunction of proteostasis significantly increases ER stress and is associated with neuronal degeneration (Scheper and Hoozemans, 2015).

The ER is found in close physical and functional connection with mitochondria and numerous lines of evidence indicate that mitochondrial dysfunction is involved in ALS. Mitochondria are the main source of cellular energy via oxidative phosphorylation and, through physical and functional interaction with the ER, they both contribute to common essential functions such as calcium homeostasis and lipid biosynthesis. Abnormalities in mitochondrial morphology have been observed in tissue from patients affected by sporadic or familial ALS, and in cellular and animal models, with defects in mitochondrial transport and morphology first demonstrated in cultured primary neurons harboring ALS causing mutations (De Vos et al., 2007; Magrane et al., 2009, 2012; Song et al., 2013). While the majority of early studies linking mitochondrial dysfunction with ALS were based on SOD1 models, it has become evident in the last decade that both functional and morphological defects in mitochondria are found in other familial ALS cases, particularly those due to mutations in TARDBP or C9ORF72.

Here we discuss recent evidence for ER and mitochondrial dysfunction associated with C9ORF72 and TARDBP mutations in the context of underlying gain of function vs. loss of function mechanisms.

## Calcium Signaling Between ER and Mitochondria

The ER takes part in multiple cellular functions, including calcium (Ca^2+^) homeostasis, lipid and protein biosynthesis, protein folding, post translational modification and regulation of gene expression (Yoshida, 2007; Eden, 2016) reviewed in Hetz and Saxena (2017). A multitude of studies suggest that disruptions in ER proteostasis and crosstalk with mitochondria can result in neuronal degeneration and motor neurons are highly susceptible to perturbations in these pathways.

One of the main functions of the ER is to store high levels of Ca^2+^, which regulate functions within the ER as well as other critical cellular functions by modulating its release into the cytosol. Ca^2+^ ions are actively transported into the ER against the gradient by the sarco/endoplasmic reticulum Ca^2+^-ATPase (SERCA) pump, and sequestered by Ca^2+^-binding proteins present in the ER. Intraluminal levels of Ca^2+^ in the ER regulate the activity of Ca^2+^-binding chaperones, such as GRP78/BiP, GRP94 and protein-disulphide isomerase (PDI) (Coe and Michalak, 2009). In addition, GRP78/BiP is also a regulator of the Unfolded Protein Response (UPR). It is involved in the folding, assembly and translocation of newly synthesized proteins, and its association with peptides is dependent on a high Ca^2+^ concentration (Vogel et al., 1990). Calnexin and calreticulin are both Ca^2+^ binding ER chaperones involved in the quality control process where they promote proper folding of nascent proteins in a Ca^2+^-dependent manner (Prell et al., 2013). These chaperones can also act as high-capacity Ca^2+^ stores at the mitochondria-associated membrane when Ca^2+^ is transferred to the mitochondria and genetic ablation of calreticulin was found to accelerate muscle denervation in ALS (Bernard-Marissal et al., 2015b).

## Calcium Miscommunication Between ER and Mitochondria in ALS

**Figure 1:**
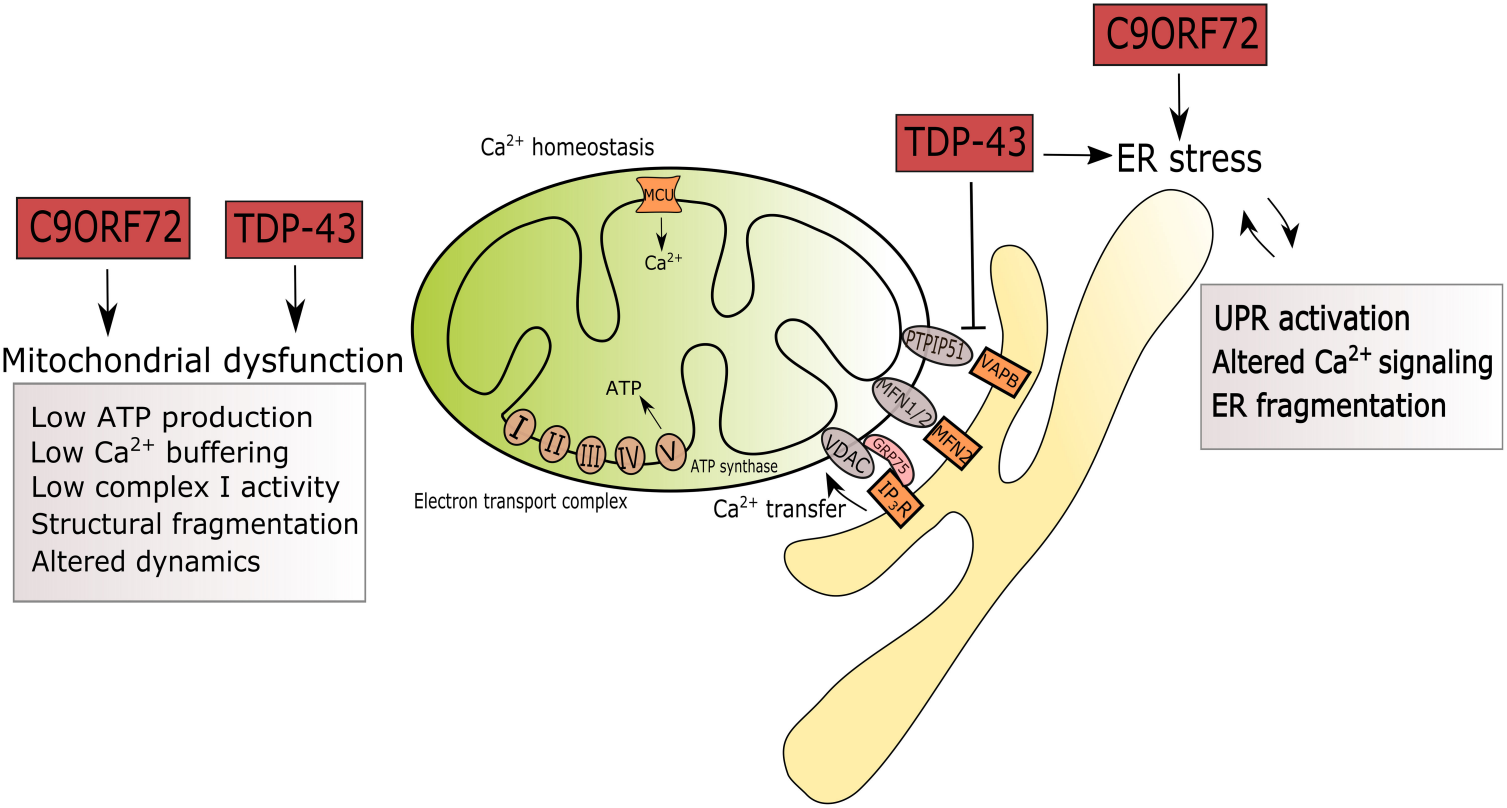
Summary of ER and mitochondrial alterations induced by mutations in C9ORF72 and TARDBP in the context of ALS. ALS-associated mutations in C9ORF72 and TARDBP are triggers of ER stress by UPR activation, altered Ca^2+^ signaling and fragmentation of the ER network. Mutant TDP-43 disrupts the tethering of PTPIP5 and VAPB. In mitochondria, mutations in C9ORF72 and TARDBP associate with reduced ATP production, Ca^2+^ signaling, complex I activity, leading to structural fragmentation and reduced mitochondrial transport in axons.

Calcium miscommunication between the ER and mitochondria has recently emerged as a major factor in loss of Ca^2+^ homeostasis in ALS. The membranes of the ER and mitochondria are closely connected at contact sites called mitochondria-associated ER membranes (MAM). The membranes of the two organelles are tethered by interactions between several protein complexes: Mitofusin 1 and 2 (MFN1/2); IP_3_R and VDAC via GRP75; and VAPB binding to protein tyrosine phosphatase-interacting protein 51 (PTPIP51) on the outer mitochondrial membrane (reviewed in Lau et al., 2018) (Figure 1). Disruptions in the interactions between these tethering complexes have been reported in several studies in SOD1, TDP-43 and FUS-related ALS (Stoica et al., 2014, 2016).

Calcium uptake in the mitochondria is a finely tuned event with profound importance both for overall cellular homeostasis and for normal mitochondrial function. Mitochondrial Ca^2+^ uptake is dependent on mitochondrial membrane potential, which is developed and maintained by mitochondrial respiration, via the electron transport chain to reduce molecular oxygen and pump out protons. Four enzymatic complexes form the electron transport chain: NADH and succinate dehydrogenases (complex I and II), ubiquinone-cytochrome *c* reductase (complex III), and cytochrome *c* oxidase (complex IV). The proton electrochemical potential drives ATP synthase to produce energy and several mitochondrial dehydrogenases are Ca^2+^ dependent (Griffiths and Rutter, 2009).

Cytosolic Ca^2+^ is buffered by mitochondria through the mitochondrial calcium uniporter (MCU), a transmembrane protein that sits on the inner mitochondrial membrane in close connection with the ER membrane (Figure 1). Its activity is regulated by the EF-hand domain-containing proteins mitochondrial calcium uptake (MICU) 1 and 2 (Perocchi et al., 2010; Plovanich et al., 2013). These regulators activate or inhibit MCU by sensing the Ca^2+^ concentration in the intermembrane space (Patron et al., 2014).

RNA sequencing of iPS-derived motor neurons from patients with mutations in C9ORF72 and TDP-43 has revealed an altered balance between these two gatekeepers in ALS (Dafinca et al., 2020). In models of TDP-43 and FUS, an impairment in the communication between ER and mitochondria leads to a reduction in Ca^2+^ uptake in the mitochondria and a subsequent rise in cytosolic Ca^2+^ which may activate cellular death pathways (Stoica et al., 2014, 2016). Consistent with these results, low mitochondrial Ca^2+^ uptake was reported in C9ORF72 and TDP-43^M337V^ iPS-derived motor neurons from ALS patients, which contributed to increased neuronal death (Dafinca et al., 2020).

## UPR Activation in ALS

The UPR is activated by three stress sensors: inositol-requiring transmembrane kinase/endonuclease (IRE1), activating transcription factor 6 (ATF6) and PKR-like ER kinase (PERK). Once activated by cleavage in the ER, ATF6 translocates to the nucleus, where it controls the transcription of target genes related to protein folding and quality control (Haze et al., 1999). IRE1 initiates splicing of the transcription factor X-Box-Binding protein 1 (XBP1), which converts it into an activator of genes responsible for protein folding, quality control and secretion of ER-associated degradation proteins (ERAD) (Calfon et al., 2002; Acosta-Alvear et al., 2007). PERK reduces protein translation in the ER by phosphorylation of the eukaryotic initiation factor 2α (eIF2α), decreasing the potential burden of misfolded proteins (Harding et al., 1999). Furthermore, eIF2α activates ATF4 driving the expression of a cascade of UPR-targeted genes responsible for protein folding, autophagy and apoptosis (Tabas and Ron, 2011). Eventually, the UPR triggers attenuation of general translation and enhanced expression of genes encoding chaperones, folding enzymes, and ERAD proteins. A failure to restore ER homeostasis, leads to activation of apoptotic pathways (Malhotra and Kaufman, 2007; Krebs et al., 2015).

Evidence for the relevance of this pathway to ALS comes from studies in human post-mortem spinal cords from sporadic or familial ALS patients, where the expression of UPR pathway is significantly increased (Ilieva et al., 2007; Atkin et al., 2008; Hetz et al., 2009; Ito et al., 2009; Sasaki, 2010). Structural alterations indicative of ER stress, such as fragmentation, have also been described in the anterior horn of the spinal cord in ALS and several chaperones involved in the ER-stress response have been detected in the cerebrospinal fluid of sporadic ALS patients (Oyanagi et al., 2008; Sasaki, 2010; Vijayalakshmi et al., 2011).

Transcriptome profiles of C9ORF72-ALS human cerebellum and frontal cortex indicate activation of UPR genes as a signature of pathology, suggesting activation of ER stress (Prudencio et al., 2015). We reported increased ER stress in iPSC-derived MNs from patients with C9ORF72 mutations, followed by reduced mitochondrial membrane potential and altered mitochondrial morphology (Dafinca et al., 2016). The cells also show evidence of oxidative stress, with stress granule formation and activation of apoptosis. RNA sequencing of primary mouse neurons expressing poly(PR) identified upregulation of genes involved in ER stress, in particular the transcription factor ATF4, indicating that poly(PR) activates the UPR (Kramer et al., 2018). This in itself is reported to upregulate RAN translation, driving further production of DPRs and becoming a feed-forward loop (Zhang et al., 2014). In a different study, expression of synthetic poly(PR) induced ER stress and inhibition of the UPR increased cell survival (Wang et al., 2019). Consistent with these reports, RAN translation of the G_4_C_2_ expansion was also found to be enhanced by ER stress and overexpression of the repeats impaired global translation, while increasing the formation of stress granules in an eIF2α-dependent manner (Green et al., 2017). In a recent post-mortem study in C9-FTD patients, overall levels of pPERK and peIF2α were higher in the hippocampus of patients and they correlated with the presence of dipeptide pathology (Gami-Patel et al., 2020). In an earlier study, downregulation of PERK in the ER was shown to improve mitochondrial Ca^2+^ dynamics and restore mitochondrial elongation, highlighting the connection between UPR and mitochondrial function (Munoz et al., 2013). In cortical and spinal motor neurons from a C9ORF72 model with (G4C2)_188_ repeats, excitotoxic stress and optogenetic neuronal stimulation act as promoters of RAN translation and ER stress response, leading to increased phosphorylation of eIF2α (Westergard et al., 2019).

Cytoplasmic aggregation of TDP-43, the pathological hallmark of ALS, may be driven by activation of ER stress in motor neurons (Ayala et al., 2011; Suzuki and Matsuoka, 2012). Pharmacological induction of ER stress in neuroblastoma cells leads to TDP-43 mislocalisation and cleavage, and C-terminal fragments colocalise with PDI, potentially interfering with ER function (Walker et al., 2013). Aggregation of TDP-43 could further contribute to increasing levels of ER stress and subsequent activation of apoptosis (Suzuki et al., 2011). Moreover, ALS mutations in TDP-43 (G294A, A315T, Q331K, M337V, N390D, D169G) were shown to cause UPR upregulation in a Neuro2A cell model (Walker et al., 2013; Wang et al., 2015).

Mechanistic studies demonstrated that ER stress led to casein kinase 1-dependent phosphorylation of TDP-43, followed by cytosolic aggregation (Nonaka et al., 2016; Hicks et al., 2020). Similar conclusions were reached in mutant FUS models, which showed ER stress and PDI positive aggregates (Farg et al., 2012). Both wild-type and ALS-mutant FUS and TDP-43 perturbed ER–mitochondria associations, accompanied by changes to the VAPB–PTPIP51 interaction and abnormal Ca^2+^ signaling between the two organelles (Stoica et al., 2014).

## Deficient Mitochondrial Bioenergetics in ALS

In addition to a role in Ca^2+^ buffering, mitochondria primarily act as a source of energy for the majority of cellular processes. Functional mitochondrial changes, such as membrane hyperpolarisation, increased ATP production and respiration are detected in C9ORF72 patient fibroblasts, along with morphological changes, such as the frequent presence of mixed populations of elongated, short mitochondria (Onesto et al., 2016). In the same study, TDP-43^A382T^ fibroblasts showed a fragmented mitochondrial network along with decreased membrane potential (Onesto et al., 2016). Our group has reported reduced membrane potential in motor neurons derived from iPS cells of C9ORF72 ALS patients and reduced mitochondrial Ca^2+^ buffering capacity in both C9ORF72 and TDP-43^M337V^ iPS-MNs from ALS patients (Dafinca et al., 2016, 2020). Furthermore, a recent study demonstrated abnormalities in the electron chain machinery in human iPS-derived MNs from C9ORF72 patients, where low basal respiration and maximal mitochondrial respiration were detected (Mehta et al., 2021). A reduction in bioenergetics in C9ORF72 iPS-derived motor neurons was correlated with lowered expression of complexes I and IV of the mitochondrial electron transport chain. Consistent with these findings, Wang et al. recently showed that C9ORF72 acts as a mitochondrial-inner-membrane-associated protein that regulates oxidative phosphorylation by stabilizing TIMMDC1, an essential component for the assembly of mitochondrial complex I (Wang et al., 2021). In their study, they also demonstrate that C9ORF72 happloinsufficiency and loss of function leads to a reduction in mitochondrial complex I activity in patient-derived neurons from C9ORF72-ALS.

The most convincing direct link between C9ORF72 and mitochondrial dysfunction has been demonstrated so far by overexpression of poly-dipeptides. Arguing for a toxic gain of function, cellular and animal models overexpressing poly-dipeptides, in particular poly(GR), have consistently shown mitochondrial alterations. Patient motor neurons derived from C9ORF72 ALS iPS cells show upregulation of the p53 pathway, high levels of DNA damage during long-term culture, followed by production of reactive oxygen species and increased mitochondrial potential (Lopez-Gonzalez et al., 2016). An interactome analysis of poly(GR) showed an abundance of mitochondrial ribosomal proteins, indicating preferential binding to the mitochondria where it is likely to induce oxidative stress. Poly(GR) expression in healthy neurons recapitulated these phenotypes. In a mouse model expressing poly(GR)_80_, compromised mitochondrial morphology was also detected with preferential binding of the dipeptide to the complex V subunit of the mitochondrial ATP-synthase (ATP5A1), inducing its ubiquitination and degradation, which is consistent with reduced levels of ATP5A1 in patient brains (Choi et al., 2019). Interestingly, poly(GR) has been suggested to act as a mitochondrial targeting signal and it can be translated in close proximity to the mitochondrial surface. Frequent stalling of its translation triggers ribosome-associated quality control and C-terminal extension which leads to potentially toxic aggregations of poly(GR) on mitochondria (Li et al., 2020).

Mitochondria have also recently emerged as a target of TDP-43 (Magrane et al., 2014; Onesto et al., 2016; Wang et al., 2016, 2017; Izumikawa et al., 2017; Davis et al., 2018; Gautam et al., 2019). Abnormal accumulations of mitochondria have been described in spinal cord motor neurons of mutant TDP-43 transgenic mice and overexpression of mutant or wild-type TDP-43 in cultured motor neurons triggered similar mitochondrial morphology and transport abnormalities to those found in SOD1 mice (Shan et al., 2010; Wang et al., 2013). Overexpression of mutant TDP-43^Q331K^ and TDP-43^M337V^ in neuroblastoma cells and in primary motor neurons leads to mitochondrial depolarisation (Hong et al., 2012; Lu et al., 2012; Wang et al., 2013). Furthermore, in TDP-43^G298S^ and TDP-43^A382T^ patient fibroblasts, complex I activity was decreased, along with reduced ATP levels and oxygen consumption (Wang et al., 2016).

RNA sequencing of the axonal compartment in motor neurons of a TDP-43 knockdown mouse revealed dysregulation of many transcripts relevant for mitochondrial function and translation (Briese et al., 2020). Of note, mitochondrial ATP synthase beta-subunit (ATP5B) was downregulated, which has previously been shown to bind to FUS in cellular and animal ALS models (Deng et al., 2018). In this study, TDP-43 knockdown led to fewer intact mitochondria in axons and reduced mitochondrial potential compared to healthy motor neurons. In a recent report, aggregated TDP-43 with ALS-associated mutations was shown to bind to and sequester a subset of nuclear encoded mitochondrial DNA, including ATP5B, while increasing expression of a different subset of mitochondrial DNA and thereby inducing a global imbalance in the mitochondria (Zuo et al., 2021). The data available linking TDP-43 mutations and mitochondrial deficiencies indicate that mitochondrial dysfunctions can occur both as a result of toxic gain of function, by direct interaction, or by loss of function, possibly at the transcriptional level.

## Mitochondrial Transport Defects in ALS Motor Neurons

The proper distribution of mitochondria in neurons is essential for healthy neuronal function and this is supported by the fact that pathology in neurodegenerative diseases often correlates with defects in mitochondrial intracellular localization (Chang et al., 2006; Rui et al., 2006; Schon and Przedborski, 2011; Reddy et al., 2012). Mitochondria provide the ATP necessary to actively transport mRNAs, proteins and organelles throughout the cells, in addition to its role in Ca^2+^ buffering and metabolite synthesis (Rui et al., 2006). This distribution is coordinated by microtubule-based transport mediated mainly by the motor proteins kinesin-1 and dynein, along with their adaptors (Schnapp and Reese, 1989; Pilling et al., 2006). Interestingly, defects in several MAM-associated proteins (MFN2, VAPB, SIGMAR1) were shown to impair axonal transport of mitochondria either by interfering with the attachment of mitochondria to motor proteins, or through an increase in cytosolic Ca^2+^ levels that leads to reduced anterograde/retrograde transport (Wang and Schwarz, 2009; Misko et al., 2012; Morotz et al., 2012; Bernard-Marissal et al., 2015a).

While defects in axonal transport have long been associated with ALS, evidence of mitochondrial transport deficits in C9ORF72 models has only recently emerged. In iPS-derived MNs from C9ORF72 patients, fast axonal transport of mitochondria was found to be impaired, with no differences reported in either axonal mitochondrial counts or transcript levels of mitochondrial tRNA (Mehta et al., 2021). In this study, a major contributing factor to the deficit in transport was a reduction in basal and maximal mitochondrial respiration and PGC1α overexpression, which increases mitochondrial biogenesis, was able to improve the transport deficit.

In a TDP-43^A315T^ mutant mouse, the earliest disease-related event observed was a reduction in retrograde mitochondrial axonal transport, which later led to accumulation of mitochondria in axon terminals and fragmentation (Magrane et al., 2014). Both SOD1 and TDP-43 have been found to bind to the mitochondrial outer membrane, suggesting that they may physically interfere with mitochondria and potentially impair its transport (Vande Velde et al., 2008; Wang et al., 2013).

## Implications of ER-Mitochondria Crosstalk for Synaptic Transmission

Synaptic transmission is an essential neuronal process and dysfunctional synapses are a major feature of several neurodegenerative disorders, including ALS. A recent study demonstrated that synaptic activity increases ER-mitochondria contacts and, conversely, loss of ER-mitochondria tethering mediated by VAPB-PTPIP51 leads to reduction of synaptic transmission (Gomez-Suaga et al., 2019). Recycling of synaptic vesicles is an energetically-demanding process which is under the tight control of Ca^2+^ signaling and the loss of connectivity between the ER and mitochondria may lead to impairment in synaptic transmission through a loss of Ca^2+^ communication and a reduction in ATP production (Brini et al., 2014). This highlights the importance of ER-mitochondria connectivity and how its disruption in ALS motor neurons may have widespread detrimental consequences on critical neuronal functions, ultimately leading to neuronal death.

## Conclusions

Despite genetic heterogeneity, functional decline at the cellular level appears to follow similar pathways in ALS. A valuable therapeutic target would therefore be an upstream event that occurs in the majority of affected ALS neurons and recent evidence points to ER stress and mitochondrial dysfunction as a potential nodal point in ALS pathology which can be targeted by drugs. Since TDP-43 pathology is found in 98% of ALS cases, including in C9ORF72-related ALS, critically evaluating the connection between TDP-43 pathology, ER-stress and mitochondrial dysfunction is important for identifying therapeutic targets.

Studies in which the ER stress response has been manipulated in an attempt to ameliorate ALS phenotypes are promising but inconclusive. Inhibition of eIF2α phosphatase reduced the phenotypes in TDP-43 mutant animal models (Vaccaro et al., 2013). However, neither genetic inhibition of the UPR via ablation of PERK, nor genetic UPR enhancement via ablation of GADD34, had a beneficial effect in mutant SOD1 mice (Dzhashiashvili et al., 2019). More recent studies in SOD1-iPSC and mouse models demonstrated that MNs are more sensitive to ER stress and identified a number of modifiers, including TUDCA, a bile acid derivative which is currently undergoing clinical trials in ALS (Thams et al., 2019; Paganoni et al., 2020).

Most gain of function studies in C9ORF72 have used models expressing one of 5 poly-dipeptides that can result from RAN translation, with the majority focusing on the arginine-rich dipeptides (polyGR or polyPR). These dipeptides were shown to be toxic in various cellular and animal models, but the reliance on overexpression should prompt caution in interpreting these studies as supporting a disease mechanism mediated by mitochondrial dysfunction. Models which activate cell death pathways for whatever reason will always involve mitochondria as an active player, and this may be secondary and non-specific. It is therefore imperative to use models expressing poly-dipeptides at physiological levels, either with an inducible system or by using smaller repeat sizes under controlled expression to avoid activation of non-specific stress responses and apoptosis.

While most studies indicate that mitochondrial dysfunction occurs as a toxic gain of function, e.g., through poly(GR), C-terminal cleaved TDP-43, or TDP-43 aggregation, there is also evidence that loss of normal TDP-43 function can induce mitochondrial dysfunction These mechanisms are not exclusive and could converge to initiate the dysfunction observed in ALS motor neurons. A loss of normal TDP-43 from the nucleus could negatively affect the transcription of mitochondrial proteins, while a toxic gain of function of cleaved TDP-43 in the cytoplasm may interfere with mitochondrial function. Given recent evidence from animal and cellular models of C9ORF72, mitochondrial dysfunction may be mediated by TDP-43 pathology which becomes the driver of mitochondrial deficits and ER stress in combination with arginine rich poly-dipeptides that associate directly with these organelles.

In summary, evidence is growing that deficient interactions between ER and mitochondria are involved in neurodegeneration, including in ALS. In support of this hypothesis, mutations in several proteins involved in the communication between ER and mitochondria are associated with genetic forms of ALS (VAPB, Sigma1R) (Nishimura et al., 2004; De Vos et al., 2012). An interactome study performed in neuronal cells identified C9ORF72 enrichment in the mitochondrial fraction due to increased interaction with members of the mitochondrial outer membrane (Blokhuis et al., 2016). The possibility of C9ORF72 directly interacting with the function of MAMs remains to be explored. Due to their close functional and physical connection, a better understanding of disease mechanisms in ALS could be achieved by studying the ER and mitochondria as a functional unit, rather than separately. Irrespective of how cellular stress originates in MNs, it is clear that modulating the ER stress response and mitochondrial dysfunction in MNs are promising therapeutic avenues for ALS, whether sporadic or familial.

## Author Contributions

The article was conceived, planned, revised by KT and RD. RD was responsible for the first draft with contributions from PB. All authors contributed to the article and approved the submitted version.

## Conflict of Interest

The authors declare that the research was conducted in the absence of any commercial or financial relationships that could be construed as a potential conflict of interest.

## References

[B1] Acosta-Alvear D. Zhou Y. Blais A. Tsikitis M. Lents N. H. Arias C.. (2007). XBP1 controls diverse cell type- and condition-specific transcriptional regulatory networks. Mol. Cell 27, 53–66. 10.1016/j.molcel.2007.06.011 17612490

[B2] Atkin J. D. Farg M. A. Walker A. K. McLean C. Tomas D. Horne M. K. (2008). Endoplasmic reticulum stress and induction of the unfolded protein response in human sporadic amyotrophic lateral sclerosis. Neurobiol. Dis. 30, 400–407. 10.1016/j.nbd.2008.02.009 18440237

[B3] Ayala V. Granado-Serrano A. B. Cacabelos D. Naudi A. Ilieva E. V. Boada J.. (2011). Cell stress induces TDP-43 pathological changes associated with ERK1/2 dysfunction: implications in ALS. Acta Neuropathol. 122, 259–270. 10.1007/s00401-011-0850-y 21706176

[B4] Bernard-Marissal N. Medard J. J. Azzedine H. Chrast R. (2015a). Dysfunction in endoplasmic reticulum-mitochondria crosstalk underlies SIGMAR1 loss of function mediated motor neuron degeneration. Brain 138, 875–890. 10.1093/brain/awv008 25678561

[B5] Bernard-Marissal N. Sunyach C. Marissal T. Raoul C. Pettmann B. (2015b). Calreticulin levels determine onset of early muscle denervation by fast motoneurons of ALS model mice. Neurobiol. Dis. 73, 130–136. 10.1016/j.nbd.2014.09.009 25277755

[B6] Blokhuis A. M. Koppers M. Groen E. J. N. van den Heuvel D. M. A. Dini Modigliani S. Anink J. J.. (2016). Comparative interactomics analysis of different ALS-associated proteins identifies converging molecular pathways. Acta Neuropathol. 132, 175–196. 10.1007/s00401-016-1575-8 27164932 PMC4947123

[B7] Briese M. Saal-Bauernschubert L. Luningschror P. Moradi M. Dombert B. Surrey V.. (2020). Loss of Tdp-43 disrupts the axonal transcriptome of motoneurons accompanied by impaired axonal translation and mitochondria function. Acta Neuropathol. Commun. 8:116. 10.1186/s40478-020-00987-6 32709255 PMC7379803

[B8] Brini M. Cali T. Ottolini D. Carafoli E. (2014). Neuronal calcium signaling: function and dysfunction. Cell Mol. Life Sci. 71, 2787–2814. 10.1007/s00018-013-1550-7 24442513 PMC11113927

[B9] Calfon M. Zeng H. Urano F. Till J. H. Hubbard S. R. Harding H. P.. (2002). IRE1 couples endoplasmic reticulum load to secretory capacity by processing the XBP-1 mRNA. Nature 415, 92–96. 10.1038/415092a 11780124

[B10] Chang D. T. Rintoul G. L. Pandipati S. Reynolds I. J. (2006). Mutant huntingtin aggregates impair mitochondrial movement and trafficking in cortical neurons. Neurobiol. Dis. 22, 388–400. 10.1016/j.nbd.2005.12.007 16473015

[B11] Choi S. Y. Lopez-Gonzalez R. Krishnan G. Phillips H. L. Li A. N. Seeley W. W.. (2019). C9ORF72-ALS/FTD-associated poly(GR) binds Atp5a1 and compromises mitochondrial function *in vivo*. Nat. Neurosci. 22, 851–862. 10.1038/s41593-019-0397-0 31086314 PMC6800116

[B12] Coe H. Michalak M. (2009). Calcium binding chaperones of the endoplasmic reticulum. Gen. Physiol. Biophys. 28, F96–F103. 20093733

[B13] Dafinca R. Barbagallo P. Farrimond L. Candalija A. Scaber J. Ababneh N. A.. (2020). Impairment of mitochondrial calcium buffering links mutations in C9ORF72 and TARDBP in iPS-derived motor neurons from patients with ALS/FTD. Stem Cell Rep. 14, 892–908. 10.1016/j.stemcr.2020.03.023 32330447 PMC7220989

[B14] Dafinca R. Scaber J. Ababneh N. Lalic T. Weir G. Christian H.. (2016). C9orf72 hexanucleotide expansions are associated with altered endoplasmic reticulum calcium homeostasis and stress granule formation in induced pluripotent stem cell-derived neurons from patients with amyotrophic lateral sclerosis and frontotemporal dementia. Stem Cells 34, 2063–2078. 10.1002/stem.2388 27097283 PMC4979662

[B15] Davis S. A. Itaman S. Khalid-Janney C. M. Sherard J. A. Dowell J. A. Cairns N. J.. (2018). TDP-43 interacts with mitochondrial proteins critical for mitophagy and mitochondrial dynamics. Neurosci. Lett. 678, 8–15. 10.1016/j.neulet.2018.04.053 29715546 PMC5975202

[B16] De Vos K. J. Chapman A. L. Tennant M. E. Manser C. Tudor E. L. Lau K. F.. (2007). Familial amyotrophic lateral sclerosis-linked SOD1 mutants perturb fast axonal transport to reduce axonal mitochondria content. Hum. Mol. Genet. 16, 2720–2728. 10.1093/hmg/ddm226 17725983 PMC4516806

[B17] De Vos K. J. Morotz G. M. Stoica R. Tudor E. L. Lau K. F. Ackerley S.. (2012). VAPB interacts with the mitochondrial protein PTPIP51 to regulate calcium homeostasis. Hum. Mol. Genet. 21, 1299–1311. 10.1093/hmg/ddr559 22131369 PMC3284118

[B18] DeJesus-Hernandez M. Mackenzie I. R. Boeve B. F. Boxer A. L. Baker M. Rutherford N. J.. (2011). Expanded GGGGCC hexanucleotide repeat in noncoding region of C9ORF72 causes chromosome 9p-linked FTD and ALS. Neuron 72, 245–256. 10.1016/j.neuron.2011.09.011 21944778 PMC3202986

[B19] Deng J. Wang P. Chen X. Cheng H. Liu J. Fushimi K.. (2018). FUS interacts with ATP synthase beta subunit and induces mitochondrial unfolded protein response in cellular and animal models. Proc. Natl. Acad. Sci. U. S. A. 115, E9678–E9686. 10.1073/pnas.1806655115 30249657 PMC6187197

[B20] Dzhashiashvili Y. Monckton C. P. Shah H. S. Kunjamma R. B. Popko B. (2019). The UPR-PERK pathway is not a promising therapeutic target for mutant SOD1-induced ALS. Neurobiol. Dis. 127, 527–544. 10.1016/j.nbd.2019.03.024 30923003 PMC6588429

[B21] Eden E. R. (2016). The formation and function of ER-endosome membrane contact sites. Biochim. Biophys. Acta 1861, 874–879. 10.1016/j.bbalip.2016.01.020 26898183 PMC4917889

[B22] Farg M. A. Soo K. Y. Walker A. K. Pham H. Orian J. Horne M. K.. (2012). Mutant FUS induces endoplasmic reticulum stress in amyotrophic lateral sclerosis and interacts with protein disulfide-isomerase. Neurobiol. Aging 33, 2855–2868. 10.1016/j.neurobiolaging.2012.02.009 22459602

[B23] Gami-Patel P. van Dijken I. Meeter L. H. Melhem S. Morrema T. H. J. Scheper W.. (2020). Unfolded protein response activation in C9orf72 frontotemporal dementia is associated with dipeptide pathology and granulovacuolar degeneration in granule cells. Brain Pathol. 31, pp 163–173. 10.1111/bpa.12894 32865835 PMC7891436

[B24] Gautam M. Jara J. H. Kocak N. Rylaarsdam L. E. Kim K. D. Bigio E. H.. (2019). Mitochondria, ER, and nuclear membrane defects reveal early mechanisms for upper motor neuron vulnerability with respect to TDP-43 pathology. Acta Neuropathol. 137, 47–69. 10.1007/s00401-018-1934-8 30450515 PMC6339587

[B25] Gomez-Suaga P. Perez-Nievas B. G. Glennon E. B. Lau D. H. W. Paillusson S. Morotz G. M.. (2019). The VAPB-PTPIP51 endoplasmic reticulum-mitochondria tethering proteins are present in neuronal synapses and regulate synaptic activity. Acta Neuropathol. Commun. 7:35. 10.1186/s40478-019-0688-4 30841933 PMC6402140

[B26] Green K. M. Glineburg M. R. Kearse M. G. Flores B. N. Linsalata A. E. Fedak S. J.. (2017). RAN translation at C9orf72-associated repeat expansions is selectively enhanced by the integrated stress response. Nat. Commun. 8:2005. 10.1038/s41467-017-02200-0 29222490 PMC5722904

[B27] Griffiths E. J. Rutter G. A. (2009). Mitochondrial calcium as a key regulator of mitochondrial ATP production in mammalian cells. Biochim. Biophys. Acta 1787, 1324–1333. 10.1016/j.bbabio.2009.01.019 19366607

[B28] Harding H. P. Zhang Y. Ron D. (1999). Protein translation and folding are coupled by an endoplasmic-reticulum-resident kinase. Nature 397, 271–274. 10.1038/16729 9930704

[B29] Haze K. Yoshida H. Yanagi H. Yura T. Mori K. (1999). Mammalian transcription factor ATF6 is synthesized as a transmembrane protein and activated by proteolysis in response to endoplasmic reticulum stress. Mol. Biol. Cell. 10, 3787–3799. 10.1091/mbc.10.11.3787 10564271 PMC25679

[B30] Hetz C. Saxena S. (2017). ER stress and the unfolded protein response in neurodegeneration. Nat. Rev. Neurol. 13, 477–491. 10.1038/nrneurol.2017.99 28731040

[B31] Hetz C. Thielen P. Matus S. Nassif M. Court F. Kiffin R.. (2009). XBP-1 deficiency in the nervous system protects against amyotrophic lateral sclerosis by increasing autophagy. Genes Dev. 23, 2294–2306. 10.1101/gad.1830709 19762508 PMC2758741

[B32] Hicks D. A. Cross L. L. Williamson R. Rattray M. (2020). Endoplasmic reticulum stress signalling induces casein kinase 1-dependent formation of cytosolic TDP-43 inclusions in motor neuron-like cells. Neurochem. Res. 45, 1354–1364. 10.1007/s11064-019-02832-2 31280399 PMC7260270

[B33] Hong K. Li Y. Duan W. Guo Y. Jiang H. Li W.. (2012). Full-length TDP-43 and its C-terminal fragments activate mitophagy in NSC34 cell line. Neurosci. Lett. 530, 144–149. 10.1016/j.neulet.2012.10.003 23063673

[B34] Ilieva E. V. Ayala V. Jove M. Dalfo E. Cacabelos D. Povedano M.. (2007). Oxidative and endoplasmic reticulum stress interplay in sporadic amyotrophic lateral sclerosis. Brain 130, 3111–3123. 10.1093/brain/awm190 17716997

[B35] Ito Y. Yamada M. Tanaka H. Aida K. Tsuruma K. Shimazawa M.. (2009). Involvement of CHOP, an ER-stress apoptotic mediator, in both human sporadic ALS and ALS model mice. Neurobiol. Dis. 36, 470–476. 10.1016/j.nbd.2009.08.013 19733664

[B36] Izumikawa K. Nobe Y. Yoshikawa H. Ishikawa H. Miura Y. Nakayama H.. (2017). TDP-43 stabilises the processing intermediates of mitochondrial transcripts. Sci. Rep. 7:7709. 10.1038/s41598-017-06953-y 28794432 PMC5550480

[B37] Kaushik S. Cuervo A. M. (2015). Proteostasis and aging. Nat. Med. 21, 1406–1415. 10.1038/nm.4001 26646497

[B38] Kramer N. J. Haney M. S. Morgens D. W. Jovicic A. Couthouis J. Li A.. (2018). CRISPR-Cas9 screens in human cells and primary neurons identify modifiers of C9ORF72 dipeptide-repeat-protein toxicity. Nat. Genet. 50, 603–612. 10.1038/s41588-018-0070-7 29507424 PMC5893388

[B39] Krebs J. Agellon L. B. Michalak M. (2015). Ca(2+) homeostasis and endoplasmic reticulum (ER) stress: An integrated view of calcium signaling. Biochem. Biophys. Res. Commun. 460, 114–121. 10.1016/j.bbrc.2015.02.004 25998740

[B40] Lau D. H. W. Hartopp N. Welsh N. J. Mueller S. Glennon E. B. Morotz G. M.. (2018). Disruption of ER-mitochondria signalling in fronto-temporal dementia and related amyotrophic lateral sclerosis. Cell Death Dis. 9:327. 10.1038/s41419-017-0022-7 29491392 PMC5832427

[B41] Li S. Wu Z. Tantray I. Li Y. Chen S. Dong J.. (2020). Quality-control mechanisms targeting translationally stalled and C-terminally extended poly(GR) associated with ALS/FTD. Proc. Natl. Acad. Sci. U. S. A. 117, 25104–25115. 10.1073/pnas.2005506117 32958650 PMC7547246

[B42] Lopez-Gonzalez R. Lu Y. Gendron T. F. Karydas A. Tran H. Yang D.. (2016). Poly(GR) in C9ORF72-related ALS/FTD compromises mitochondrial function and increases oxidative stress and DNA damage in iPSC-derived motor neurons. Neuron 92, 383–391. 10.1016/j.neuron.2016.09.015 27720481 PMC5111366

[B43] Lu J. Duan W. Guo Y. Jiang H. Li Z. Huang J.. (2012). Mitochondrial dysfunction in human TDP-43 transfected NSC34 cell lines and the protective effect of dimethoxy curcumin. Brain Res. Bull. 89, 185–190. 10.1016/j.brainresbull.2012.09.005 22986236

[B44] Magrane J. Cortez C. Gan W. B. Manfredi G. (2014). Abnormal mitochondrial transport and morphology are common pathological denominators in SOD1 and TDP43 ALS mouse models. Hum. Mol. Genet. 23, 1413–1424. 10.1093/hmg/ddt528 24154542 PMC3929084

[B45] Magrane J. Hervias I. Henning M. S. Damiano M. Kawamata H. Manfredi G. (2009). Mutant SOD1 in neuronal mitochondria causes toxicity and mitochondrial dynamics abnormalities. Hum. Mol. Genet. 18, 4552–4564. 10.1093/hmg/ddp421 19779023 PMC2773270

[B46] Magrane J. Sahawneh M. A. Przedborski S. Estevez A. G. Manfredi G. (2012). Mitochondrial dynamics and bioenergetic dysfunction is associated with synaptic alterations in mutant SOD1 motor neurons. J. Neurosci. 32, 229–242. 10.1523/JNEUROSCI.1233-11.2012 22219285 PMC3566782

[B47] Malhotra J. D. Kaufman R. J. (2007). The endoplasmic reticulum and the unfolded protein response. Semin. Cell Dev. Biol. 18, 716–731. 10.1016/j.semcdb.2007.09.003 18023214 PMC2706143

[B48] Mathis S. Goizet C. Soulages A. Vallat J. M. Masson G. L. (2019). Genetics of amyotrophic lateral sclerosis: a review. J. Neurol. Sci. 399, 217–226. 10.1016/j.jns.2019.02.030 30870681

[B49] McCann E. P. Henden L. Fifita J. A. Zhang K. Y. Grima N. Bauer D. C.. (2020). Evidence for polygenic and oligogenic basis of Australian sporadic amyotrophic lateral sclerosis. J. Med. Genet. 58:87–95. 10.1136/jmedgenet-2020-106866 32409511

[B50] Mehta A. R. Gregory J. M. Dando O. Carter R. N. Burr K. Nanda J.. (2021). Mitochondrial bioenergetic deficits in C9orf72 amyotrophic lateral sclerosis motor neurons cause dysfunctional axonal homeostasis. Acta Neuropathol. 141, 257–279. 10.1007/s00401-020-02252-5 33398403 PMC7847443

[B51] Misko A. L. Sasaki Y. Tuck E. Milbrandt J. Baloh R. H. (2012). Mitofusin2 mutations disrupt axonal mitochondrial positioning and promote axon degeneration. J. Neurosci. 32, 4145–4155. 10.1523/JNEUROSCI.6338-11.2012 22442078 PMC3319368

[B52] Morotz G. M. De Vos K. J. Vagnoni A. Ackerley S. Shaw C. E. Miller C. C. (2012). Amyotrophic lateral sclerosis-associated mutant VAPBP56S perturbs calcium homeostasis to disrupt axonal transport of mitochondria. Hum. Mol. Genet. 21, 1979–1988. 10.1093/hmg/dds011 22258555 PMC3315205

[B53] Munoz J. P. Ivanova S. Sanchez-Wandelmer J. Martinez-Cristobal P. Noguera E. Sancho A.. (2013). Mfn2 modulates the UPR and mitochondrial function via repression of PERK. EMBO J. 32, 2348–2361. 10.1038/emboj.2013.168 23921556 PMC3770335

[B54] Nishimura A. L. Mitne-Neto M. Silva H. C. Richieri-Costa A. Middleton S. Cascio D.. (2004). A mutation in the vesicle-trafficking protein VAPB causes late-onset spinal muscular atrophy and amyotrophic lateral sclerosis. Am. J. Hum. Genet. 75, 822–831. 10.1086/425287 15372378 PMC1182111

[B55] Nonaka T. Suzuki G. Tanaka Y. Kametani F. Hirai S. Okado H.. (2016). Phosphorylation of TAR DNA-binding protein of 43 kDa (TDP-43) by truncated casein kinase 1delta triggers mislocalization and accumulation of TDP-43. J. Biol. Chem. 291, 5473–5483. 10.1074/jbc.M115.695379 26769969 PMC4786690

[B56] Onesto E. Colombrita C. Gumina V. Borghi M. O. Dusi S. Doretti A.. (2016). Gene-specific mitochondria dysfunctions in human TARDBP and C9ORF72 fibroblasts. Acta Neuropathol. Commun. 4:47. 10.1186/s40478-016-0316-5 27151080 PMC4858818

[B57] Oyanagi K. Yamazaki M. Takahashi H. Watabe K. Wada M. Komori T.. (2008). Spinal anterior horn cells in sporadic amyotrophic lateral sclerosis show ribosomal detachment from, and cisternal distention of the rough endoplasmic reticulum. Neuropathol. Appl. Neurobiol. 34, 650–658. 10.1111/j.1365-2990.2008.00941.x 18346115

[B58] Paganoni S. Macklin E. A. Hendrix S. Berry J. D. Elliott M. A. Maiser S.. (2020). Trial of sodium phenylbutyrate-taurursodiol for amyotrophic lateral sclerosis. N. Engl. J. Med. 383, 919–930. 10.1056/NEJMoa1916945 32877582 PMC9134321

[B59] Patron M. Checchetto V. Raffaello A. Teardo E. Vecellio Reane D. Mantoan M.. (2014). MICU1 and MICU2 finely tune the mitochondrial Ca2+ uniporter by exerting opposite effects on MCU activity. Mol. Cell. 53, 726–737. 10.1016/j.molcel.2014.01.013 24560927 PMC3988891

[B60] Perocchi F. Gohil V. M. Girgis H. S. Bao X. R. McCombs J. E. Palmer A. E.. (2010). MICU1 encodes a mitochondrial EF hand protein required for Ca(2+) uptake. Nature 467, 291–296. 10.1038/nature09358 20693986 PMC2977980

[B61] Pilling A. D. Horiuchi D. Lively C. M. Saxton W. M. (2006). Kinesin-1 and Dynein are the primary motors for fast transport of mitochondria in Drosophila motor axons. Mol. Biol. Cell 17, 2057–2068. 10.1091/mbc.e05-06-0526 16467387 PMC1415296

[B62] Plovanich M. Bogorad R. L. Sancak Y. Kamer K. J. Strittmatter L. Li A. A.. (2013). MICU2, a paralog of MICU1, resides within the mitochondrial uniporter complex to regulate calcium handling. PLoS ONE 8:e55785. 10.1371/journal.pone.0055785 23409044 PMC3567112

[B63] Prell T. Lautenschlager J. Grosskreutz J. (2013). Calcium-dependent protein folding in amyotrophic lateral sclerosis. Cell Calcium 54, 132–143. 10.1016/j.ceca.2013.05.007 23764168

[B64] Prudencio M. Belzil V. V. Batra R. Ross C. A. Gendron T. F. Pregent L. J.. (2015). Distinct brain transcriptome profiles in C9orf72-associated and sporadic ALS. Nat. Neurosci. 18, 1175–1182. 10.1038/nn.4065 26192745 PMC4830686

[B65] Reddy P. H. Tripathi R. Troung Q. Tirumala K. Reddy T. P. Anekonda V.. (2012). Abnormal mitochondrial dynamics and synaptic degeneration as early events in Alzheimer's disease: implications to mitochondria-targeted antioxidant therapeutics. Biochim. Biophys. Acta 1822, 639–649. 10.1016/j.bbadis.2011.10.011 22037588 PMC3272314

[B66] Renton A. E. Majounie E. Waite A. Simon-Sanchez J. Rollinson S. Gibbs J. R.. (2011). A hexanucleotide repeat expansion in C9ORF72 is the cause of chromosome 9p21-linked ALS-FTD. Neuron 72, 257–268. 10.1016/j.neuron.2011.09.010 21944779 PMC3200438

[B67] Rui Y. Tiwari P. Xie Z. Zheng J. Q. (2006). Acute impairment of mitochondrial trafficking by beta-amyloid peptides in hippocampal neurons. J. Neurosci. 26, 10480–10487. 10.1523/JNEUROSCI.3231-06.2006 17035532 PMC6674697

[B68] Sasaki S. (2010). Endoplasmic reticulum stress in motor neurons of the spinal cord in sporadic amyotrophic lateral sclerosis. J. Neuropathol. Exp. Neurol. 69, 346–355. 10.1097/NEN.0b013e3181d44992 20448480

[B69] Scheper W. Hoozemans J. J. (2015). The unfolded protein response in neurodegenerative diseases: a neuropathological perspective. Acta Neuropathol. 130, 315–331. 10.1007/s00401-015-1462-8 26210990 PMC4541706

[B70] Schnapp B. J. Reese T. S. (1989). Dynein is the motor for retrograde axonal transport of organelles. Proc. Natl. Acad. Sci. U. S. A. 86, 1548–1552. 10.1073/pnas.86.5.1548 2466291 PMC286735

[B71] Schon E. A. Przedborski S. (2011). Mitochondria: the next (neurode)generation. Neuron 70, 1033–1053. 10.1016/j.neuron.2011.06.003 21689593 PMC3407575

[B72] Schroder M. (2008). Endoplasmic reticulum stress responses. Cell Mol. Life Sci. 65, 862–894. 10.1007/s00018-007-7383-5 18038217 PMC11131897

[B73] Shan X. Chiang P. M. Price D. L. Wong P. C. (2010). Altered distributions of Gemini of coiled bodies and mitochondria in motor neurons of TDP-43 transgenic mice. Proc. Natl. Acad. Sci. U. S. A. 107, 16325–16330. 10.1073/pnas.1003459107 20736350 PMC2941282

[B74] Song W. Song Y. Kincaid B. Bossy B. Bossy-Wetzel E. (2013). Mutant SOD1G93A triggers mitochondrial fragmentation in spinal cord motor neurons: neuroprotection by SIRT3 and PGC-1alpha. Neurobiol. Dis. 51, 72–81. 10.1016/j.nbd.2012.07.004 22819776 PMC3992938

[B75] Stoica R. De Vos K. J. Paillusson S. Mueller S. Sancho R. M. Lau K. F.. (2014). ER-mitochondria associations are regulated by the VAPB-PTPIP51 interaction and are disrupted by ALS/FTD-associated TDP-43. Nat. Commun. 5:3996. 10.1038/ncomms4996 24893131 PMC4046113

[B76] Stoica R. Paillusson S. Gomez-Suaga P. Mitchell J. C. Lau D. H. Gray E. H.. (2016). ALS/FTD-associated FUS activates GSK-3beta to disrupt the VAPB-PTPIP51 interaction and ER-mitochondria associations. EMBO Rep. 17, 1326–1342. 10.15252/embr.201541726 27418313 PMC5007559

[B77] Suzuki H. Lee K. Matsuoka M. (2011). TDP-43-induced death is associated with altered regulation of BIM and Bcl-xL and attenuated by caspase-mediated TDP-43 cleavage. J. Biol. Chem. 286, 13171–13183. 10.1074/jbc.M110.197483 21339291 PMC3075664

[B78] Suzuki H. Matsuoka M. (2012). TDP-43 toxicity is mediated by the unfolded protein response-unrelated induction of C/EBP homologous protein expression. J. Neurosci. Res. 90, 641–647. 10.1002/jnr.22777 22057717

[B79] Tabas I. Ron D. (2011). Integrating the mechanisms of apoptosis induced by endoplasmic reticulum stress. Nat. Cell Biol. 13, 184–190. 10.1038/ncb0311-184 21364565 PMC3107571

[B80] Thams S. Lowry E. R. Larraufie M. H. Spiller K. J. Li H. Williams D. J.. (2019). A stem cell-based screening platform identifies compounds that desensitize motor neurons to endoplasmic reticulum stress. Mol. Ther. 27, 87–101. 10.1016/j.ymthe.2018.10.010 30446391 PMC6318783

[B81] Vaccaro A. Patten S. A. Aggad D. Julien C. Maios C. Kabashi E.. (2013). Pharmacological reduction of ER stress protects against TDP-43 neuronal toxicity *in vivo*. Neurobiol. Dis. 55, 64–75. 10.1016/j.nbd.2013.03.015 23567652

[B82] Vande Velde C. Miller T. M. Cashman N. R. Cleveland D. W. (2008). Selective association of misfolded ALS-linked mutant SOD1 with the cytoplasmic face of mitochondria. Proc. Natl. Acad. Sci. U. S. A. 105, 4022–4027. 10.1073/pnas.0712209105 18296640 PMC2268797

[B83] Vijayalakshmi K. Alladi P. A. Ghosh S. Prasanna V. K. Sagar B. C. Nalini A.. (2011). Evidence of endoplasmic reticular stress in the spinal motor neurons exposed to CSF from sporadic amyotrophic lateral sclerosis patients. Neurobiol. Dis. 41, 695–705. 10.1016/j.nbd.2010.12.005 21168498

[B84] Vogel J. P. Misra L. M. Rose M. D. (1990). Loss of BiP/GRP78 function blocks translocation of secretory proteins in yeast. J. Cell. Biol. 110, 1885–1895. 10.1083/jcb.110.6.1885 2190988 PMC2116122

[B85] Walker A. K. Soo K. Y. Sundaramoorthy V. Parakh S. Ma Y. Farg M. A.. (2013). ALS-associated TDP-43 induces endoplasmic reticulum stress, which drives cytoplasmic TDP-43 accumulation and stress granule formation. PLoS ONE 8:e81170. 10.1371/journal.pone.0081170 24312274 PMC3843686

[B86] Wang R. Xu X. Hao Z. Zhang S. Wu D. Sun H.. (2019). Poly-PR in C9ORF72-related amyotrophic lateral sclerosis/frontotemporal dementia causes neurotoxicity by clathrin-dependent endocytosis. Neurosci. Bull. 35, 889–900. 10.1007/s12264-019-00395-4 31148094 PMC6754483

[B87] Wang T. Liu H. Itoh K. Oh S. Zhao L. Murata D.. (2021). C9orf72 regulates energy homeostasis by stabilizing mitochondrial complex I assembly. Cell. Metab. 3, 531–546. 10.1016/j.cmet.2021.01.005 33545050 PMC8579819

[B88] Wang W. Arakawa H. Wang L. Okolo O. Siedlak S. L. Jiang Y.. (2017). Motor-coordinative and cognitive dysfunction caused by mutant TDP-43 could be reversed by inhibiting its mitochondrial localization. Mol. Ther. 25, 127–139. 10.1016/j.ymthe.2016.10.013 28129109 PMC5363201

[B89] Wang W. Li L. Lin W. L. Dickson D. W. Petrucelli L. Zhang T.. (2013). The ALS disease-associated mutant TDP-43 impairs mitochondrial dynamics and function in motor neurons. Hum. Mol. Genet. 22, 4706–4719. 10.1093/hmg/ddt319 23827948 PMC3820133

[B90] Wang W. Wang L. Lu J. Siedlak S. L. Fujioka H. Liang J.. (2016). The inhibition of TDP-43 mitochondrial localization blocks its neuronal toxicity. Nat. Med. 22, 869–878. 10.1038/nm.4130 27348499 PMC4974139

[B91] Wang X. Schwarz T. L. (2009). The mechanism of Ca2+ -dependent regulation of kinesin-mediated mitochondrial motility. Cell 136, 163–174. 10.1016/j.cell.2008.11.046 19135897 PMC2768392

[B92] Wang X. Zhou S. Ding X. Ma M. Zhang J. Zhou Y.. (2015). Activation of ER stress and autophagy induced by TDP-43 A315T as pathogenic mechanism and the corresponding histological changes in skin as potential biomarker for ALS with the mutation. Int. J. Biol. Sci. 11, 1140–1149. 10.7150/ijbs.12657 26327808 PMC4551750

[B93] Westergard T. McAvoy K. Russell K. Wen X. Pang Y. Morris B.. (2019). Repeat-associated non-AUG translation in C9orf72-ALS/FTD is driven by neuronal excitation and stress. EMBO Mol. Med. 11:e9423. 10.15252/emmm.201809423 30617154 PMC6365928

[B94] Yoshida H. (2007). ER stress and diseases. FEBS J. 274, 630–658. 10.1111/j.1742-4658.2007.05639.x 17288551

[B95] Zhang Y. J. Jansen-West K. Xu Y. F. Gendron T. F. Bieniek K. F. Lin W. L.. (2014). Aggregation-prone c9FTD/ALS poly(GA) RAN-translated proteins cause neurotoxicity by inducing ER stress. Acta Neuropathol. 128, 505–524. 10.1007/s00401-014-1336-5 25173361 PMC4159567

[B96] Zuo X. Zhou J. Li Y. Wu K. Chen Z. Luo Z.. (2021). TDP-43 aggregation induced by oxidative stress causes global mitochondrial imbalance in ALS. Nat. Struct. Mol. Biol. 28, 132–142. 10.1038/s41594-020-00537-7 33398173

